# Effects of Electrospun Fibrous Membranes of PolyCaprolactone and Chitosan/Poly(Ethylene Oxide) on Mouse Acute Skin Lesions

**DOI:** 10.3390/polym12071580

**Published:** 2020-07-16

**Authors:** Flávia Cristina Zanchetta, Rafael Bergamo Trinca, Juliany Lino Gomes Silva, Jéssica da Silva Cunha Breder, Thiago Anselmo Cantarutti, Sílvio Roberto Consonni, Ângela Maria Moraes, Eliana Pereira de Araújo, Mario José Abdalla Saad, Gary G. Adams, Maria Helena Melo Lima

**Affiliations:** 1 School of Nursing, University of Campinas, Campinas CEP 13083887, Brazil; flaviac.zanchetta@gmail.com (F.C.Z.); julianyl@unicamp.br (J.L.G.S.); jecunha.silva@gmail.com (J.d.S.C.B.); biocantarutti@gmail.com (T.A.C.); pa.eliana@gmail.com (E.P.d.A.); 2 Department of Engineering of Materials and of Bioprocess, School of Chemical Engineering, University of Campinas, Campinas CEP 13083852, Brazil; rafaeltrinca@gmail.com (R.B.T.); ammoraes@unicamp.br (Â.M.M.); 3 Department of Biochemistry and Tissue Biology, Institute of Biology, University of Campinas, Campinas CEP 13083970, Brazil; consonni@unicamp.br; 4 Department of Internal Medicine, University of Campinas, Campinas CEP 13083887, Brazil; msaad@fcm.unicamp.br; 5 School of Health Sciences, Faculty of Medicine, The University of Nottingham, C Floor, South Block Link, Queen’s Medical Centre, Nottingham NG7 2HA, UK

**Keywords:** C57BL/6J mice, wound healing, wound dressing, chitosan, polycaprolactone, electrospinning

## Abstract

Polycaprolactone (PCL) is a synthetic polymer with good mechanical properties that are useful to produce biomaterials of clinical application. It can be successfully combined with chitosan, which enhances the biomaterial properties through the modulation of molecular and cellular mechanisms. The objective of this study was to evaluate the effects of the use of electrospun fibrous membranes consisting of polycaprolactone (PCL) or polycaprolactone coated with chitosan and poly(ethylene oxide) (PCL+CHI/PEO) on mouse skin lesions. Sixty four Black-57 mice were divided into PCL and PCL+CHI/PEO groups. A 1 cm^2^ lesion was made on the animals’ backs, and the membranes were sutured in place. The tissues were extracted on the 3rd, 7th, and 14th days after the lesion. The tissues were analyzed by histology with Hematoxylin and Eosin (H&E) and Sirius Red stains, morphometry, immunohistochemistry, and Western blot. On the 3rd, 6th, and 9th days after the lesion, the PCL+CHI/PEO group showed a higher wound-healing rate (WHR). On the 3 day, the PCL+CHI/PEO group showed a greater amount of inflammatory infiltrate, greater expression of proliferating cell nuclear antigen (PCNA), and smooth muscle actin (α-SMA) (*p* < 0.05) compared to the PCL group. On the 7th day after the lesion, the PCL+CHI/PEO group showed a greater amount of inflammatory infiltrate, expression of Tumor Necrosis Factor (TNF-α) and PCNA (*p* < 0.05). In addition, it showed a greater immunolabeling of Monocyte Chemoattractant Protein-1 (MCP-1) and deposition of collagen fibers compared to the PCL group. The PCL+CHI/PEO membrane modulated the increase in the inflammatory infiltrate, the expression of MCP-1, PCNA, and α-SMA in lesions of mice.

## 1. Introduction

The presence of cutaneous lesions impairs the functions exerted by the skin, which, once damaged, triggers molecular and cellular mechanisms to restore the injured area [1]. Several factors, such as diabetes mellitus, can interfere with the physiological healing process, preventing or delaying the tissue repair process [2].

For this reason, the search for new therapeutic strategies to promote a faster healing of lesions is a worldwide challenge. For this to be possible, these dressings must meet some basic principles, such as being hypoallergenic and biocompatible, providing moisture maintenance at appropriate limits, allowing gas exchange, providing thermal control, being flexible and able to adapt to the injury site, favoring cell proliferation, and protecting the injured area from contaminants, among other characteristics [3,4].

The use of chitosan-based wound coverings is a reality in the market—for instance, products such as ChitoFlex^®^ PRO, ChitoGauze^®^ PRO XR, and Celox GauzeTM [5]. Chitosan can be obtained from the deacetylation of chitin, which is found in the exoskeleton of crustaceans, insects, and mollusks and in the cell wall of fungi [6,7]. Through deacetylation, it is possible to increase the solubility of the natural polysaccharide and its technological applicability [3].

Chitosan has very attractive features, such as biodegradability, biocompatibility [8], and fluid absorption properties [7], which makes it a viable option for creating wound dressings and producing supports for tissue engineering (scaffolds) [9]. Moreover, it has hemostatic activity, and during the healing process, it offers a nonprotein matrix for tissue growth, stimulating cell proliferation and tissue organization by contributing to the proliferation of fibroblasts and increasing collagen deposition [8,10]. Regarding biological properties, it is bacteriostatic and acts against fungi and viruses [11].

Despite the numerous favorable characteristics for the treatment of lesions, chitosan has restricted and isolated use due to its low mechanical resistance, especially in hydrated states, such as exudative wounds [12,13]. In addition, its direct processing for the manufacture of fibrous dressings with high surface area is not possible due to the high viscosity of chitosan solutions. Therefore, devices that combine the properties of chitosan with those of synthetic polymers, such as polycaprolactone (PCL), can be useful for the final application of the product [14,15,16].

Polycaprolactone (PCL) is an aliphatic polyester with a semi-crystalline structure that shows high solubility in many volatile organic solvents. This polymer has higher rheological and viscoelastic properties than several synthetic polymers [17]. Furthermore, it is stable in environmental conditions, has low cost, is available in large amounts, has good mechanical resistance [14], is capable of contributing to adhesion, migration, and proliferation of fibroblasts, and is biocompatible with different types of tissues [17,18]. Additional advantages of the use of PCL for the production of wound dressings include the fact that its degradation process does not generate any toxic by-product, while its mechanical properties are compatible with those of the human skin [19,20,21].

Different processing techniques can be applied to produce biomaterials useful as wound dressings from a variety of polymers, e.g., solvent casting, thermal or solvent-induced phase inversion, additive manufacturing, and electrospinning. These techniques have been successfully applied in the production of dense and porous membranes applicable in tissue engineering and many other areas, such as composite materials, filtration membranes, and many others [22,23,24,25].

In this study, to overcome the limitations of the isolated use of both chitosan and PCL, we used the electrospinning technique to make fibrous membranes. This process consists of applying a high electrical potential between a grounded collector and a needle from which the polymeric solution is expelled in a constant flow. When the applied voltage is sufficiently high, the solution drops at the end of the needle become charged and the electrostatic repulsion counteracts the surface tension of the drop, deforming it. At a critical voltage, the liquid is expelled from the surface of the droplet and the electrostatic repulsion causes the lashing of the jets, resulting in their elongation and thinning, which takes the form of fibers that can reach nanometric dimensions when directed to the grounded collector [26].

The electrospinning process has low cost and allows the production of porous uniform films and membranes showing high surface area, porosity, and interconnectivity. The structure of the biomaterials obtained by electrospinning is capable of mimicking the extracellular matrix of different tissues, including the skin, and thus favoring the healing process. For these reasons, and due to many advances experienced in the electrospinning technology lately, the use of this process to produce material for biomedical applications has expanded significantly, contributing to the manufacture of wound dressings and scaffolds for the area of tissue engineering [27].

The use of layered micro and nanofibrous biomaterials in the area of wound dressings is quite attractive. Most of these materials are fabricated in such a way to provide desired mechanical properties such as strength and fatigue resistance as well as improved water and oxygen permeability. The presence of nanofibers in wound dressings may contribute to improving the hemostasis and absorption of exudates, while also aiding in the maintenance of adequate moisture levels in the wound environment. Several studies have demonstrated the use of nanofibers for the treatment of wounds [28,29,30]. In general, wounds are resolved by the physiological response; however, systemic or local factors can contribute to delayed healing. Therefore, the availability of wound dressings that can contribute to this process is extremely important.

In this work, to obtain more stable jets and properly stretched fibers, the chitosan solution was mixed with poly(ethylene oxide), PEO, which is a biocompatible and uncharged synthetic polymer that is widely used in the biomaterials area due to its hydrophilic and non-immunogenic behavior and also to minimize cell and protein adhesion [31,32]. PEO can associate with chitosan by hydrogen bonds, decreasing the viscosity, surface tension, and conductivity of the chitosan solution, which facilitates the polysaccharide electrospinning process, while also increasing the number of molecular intertwining [32], improving the connection of the chains and contributing to increase the stability of the chitosan (CHI) layer.

Therefore, this study aimed to compare the effects of membranes made up only of PCL to those of porous structures in the form of overlapping bilayers of polycaprolactone coated with chitosan and poly(ethylene oxide) (PCL+CHI/PEO) obtained by electrospinning in a model of acute skin lesions in mice. As previously reported [33], the design of the multilayered membrane (PCL+CHI/PEO) aimed to couple the advantageous mechanical properties of PCL to the relevant bioactive properties of chitosan when in contact with open wounds, improving thus the polysaccharide solution spinnability and the moisture of the wound bed through its mixture with PEO.

## 2. Materials and Methods

### 2.1. Materials

Chitosan 75–85% deacetylated (CHI, 50–190 kg mol^−1^), polycaprolactone (PCL, 70–90 kg mol^−1^), and poly(ethylene oxide) (PEO, 1000 kg mol^−1^) were purchased from Sigma-Aldrich Inc. (St. Louis, MO, USA) and used as received. Chloroform, methanol, and acetic acid were acquired from Synth (Diadema, SP, Brazil) and also used as received. For the preparation of solutions and washing procedures, we used deionized water.

#### Electrospun Scaffolds Preparation

Electrospun scaffolds of PCL and double layer scaffolds PCL+CHI/PEO were produced as previously reported [33]. For the production of PCL scaffolds, 20 mL of a 15 wt % PCL solution in 5:1 *v*/*v* chloroform/methanol mixture were electrospun using a needle-to-collector distance of 15 cm. We also used an applied voltage of 20 kV and a flow rate of 30 mL h^−1^. For double-layer scaffolds, 6 mL of CHI/PEO solution (CHI 2 wt % and PEO 2 wt % in a 10% *v*/*v* acetic acid solution, reaching a total polymer concentration of 4 wt %) were electrospun directly over a recently prepared PCL scaffold (needle-to-collector distance of 15 cm, an applied voltage of 25 kV, and flow rate of 2 mL h^−1^). The electrospinning time was equal to 40 min for PCL layers (20 mL of PCL solution ejected at 30 mL/h flow rate) and 3 h for CHI/PEO layers (6 mL at 2 mL/h flow rate). All chitosan-containing scaffolds were crosslinked by exposure to glutaraldehyde in a closed chamber. The electrospinning setup consisted of a high-voltage power source (positive 0–30 kV, laboratory-made), a syringe pump (infusion pump model 670T, Samtronic Ind. r Com. Ltd.a., São Paulo, Brazil), and a rotating cylindrical collector (area 37.7 × 14 cm^2^; two parallel blunt 18-gauge needles were used as nozzles).

The aspect of the membranes was analyzed by scanning electron microscopy (SEM) in a Leo 440i instrument (LEO Electron Microscopy, Cambridge, England). Samples were set on aluminum stubs using double-sided carbon adhesive tape, sputter-coated with gold (layer approximately 200 Å thick), and were examined with an accelerating voltage of 20 kV.

### 2.2. Methods

#### 2.2.1. In Vivo Studies

All procedures adopted were approved by the Ethics Committee for the Use of Animals (CEUA) of the University of Campinas (approval no. 4354-1). Sixty-four male Black 57 mice of adult age (7–8 weeks) weighing about 25 g each were obtained from the vivarium of the University of Campinas, São Paulo, Brazil. The animals were initially kept in collective cages at a temperature between 20 and 25 °C, in light-controlled conditions (12–12 h light and dark cycles), and received a standard solid diet and water ad libitum.

#### 2.2.2. Surgical Procedures

The surgical procedure was performed under general anesthesia with 180 mg/kg of ketamine hydrochloride and 8 mg/kg of xylazine hydrochloride intraperitoneally (IP). After anesthesia, trichotomy of the dorsal region was carried out with a stainless steel razor blade. To demarcate the lesion area on the dorsal midline, a 1 cm² plastic mold and a hydrographic pen were used, according to the literature [34]. The animal’s skin was removed with the aid of surgical scissors and forceps until the muscular fascia was exposed in the predefined area. The membranes, previously hydrated with 0.9% saline, were placed under the lesion area and sutured at two points on opposite sides with 7-0 Nylon thread. To control pain, tramadol hydrochloride IP (20 mg/kg) was used immediately after the surgical procedure and in the three days following the surgery [34,35,36].

#### 2.2.3. Experimental Design and Lesion Treatment

The animals were randomly distributed in individual cages and subsequently divided into two groups. Group 1 was formed by animals treated with PCL membrane and Group 2 was formed by animals treated with PCL+CHI/PEO membranes. The membranes were hydrated once a day with about 0.1 mL of 0.9% saline. Wound healing was assessed using Canon^®^ digital camera photographs (PowerShot SX400 IS, Canon Inc., Tokyo, Japan) on days 0, 3, 6, 9, 12, and 14. For standardization, the camera was fixed to a support perpendicular to the lesion 20 cm away from the animal. The images were digitalized, and the wound areas were measured using Image J^®^ software 1.49v (National Institutes of Health, Bethesda, MD USA, JAVA). The wound closure, expressed as a percentage of the initial wound area, was analyzed based on the wound-healing rate (WHR), which was calculated using the equation below:

(1) $$\mathrm{HR}=\left[\frac{\left(\mathrm{Initial}\;\mathrm{wound}\;\mathrm{area}\right)-\left(\mathrm{Area}\;\mathrm{determined}\;\mathrm{at}\;\mathrm{an}\;\mathrm{specific}\;\mathrm{time}\;\mathrm{point}\right)}{\left(\mathrm{Initial}\;\mathrm{area}\right)}\right]\times 100\%$$

#### 2.2.4. Sample Collection

The animals were anesthetized with 180 mg/kg of ketamine hydrochloride and 8 mg/kg of xylazine hydrochloride IP. After the pain and corneal reflexes ceased, an area of 4 cm^2^ was delimited around the wound with a mold, and samples were extracted on days 3, 7, and 14 after the lesion. The samples were divided into two parts: one was fixed in 4% formaldehyde for histological analysis, and the other was initially frozen in liquid nitrogen and then kept under refrigeration at −80 °C for the Western blot. At the end of the experiment, the animals were euthanized with an overdose of anesthetics.

#### 2.2.5. Histological Analysis

The samples were fixed in pieces of cork and in 4% formaldehyde (Merck) for 8 h in a cold chamber. Afterward, they were dehydrated and diaphanized by exposure to a 70% ethanol solution (overnight) twice in 95% and 100% ethanol solutions, and finally, also twice, in Xylol, each passage lasting an hour. Then, the samples were embedded in paraffin, cut (5 µm thick) in a manual rotary microtome (Zeiss^®^, Jena, Thüringer, Germany), and transferred to slides previously covered with Poly-L-lysine (Sigma^®^,St. Louis, MO, USA). The slides were stained with Hematoxylin and Eosin (H&E) [37]. Digital images of tissues were obtained using an automatic digital slide scanner (Pannoramic MIDI, 3DHISTECwH Ltd., Budapest, Hungary).

#### 2.2.6. Histomorphometric Analysis with Light Microscopy

With the aid of a reticle attached to the microscope eyepiece, using the 100× objective, the numbers of inflammatory infiltrate, fibroblasts, and blood vessels were counted in the injured area on the 3rd and 7th days after the wound was made using the H&E slides. Fifteen areas of 100 µm² each were counted within the injured region. The analyzed areas were determined following a straight line, always moving the field in a single direction, with spacing from 100 to 300 μm between two analyzed regions, maintaining five areas just below the injured epidermis, five in a lower region, and five close to the *panniculus carnosus* [38].

#### 2.2.7. Collagen Fiber Analysis

The slides with samples from the 7th and 14th days of extraction were stained with Sirius Red. The analysis was performed by birefringence under polarized light. The collagen fibers were determined according to their birefringence pattern (greenish/greenish-yellow or orange/reddish-orange) and morphological appearance (wavy or stretched, thin or thick, short or long). The slides were visualized using a microscope (model BX51, Olympus Corporation, Center Valley, PA, USA) with a 10× objective, photographed with an Olympus DP72 camera, and quantified using ImagePro Plus^®^ (Version 6.0). The results were expressed in terms of semi-quantitative and qualitative observation of the fibers based on their aspect [39,40].

#### 2.2.8. Immunohistochemical Analysis

Sections of 5 µm of tissue from days 3, 7, and 14 were cut and transferred to glass slides coated with silane (Starfrost^®^, Craftsman’s Way, Lowestoft, UK). Then, they were incubated with primary antibodies specific for markers: proliferating cell nuclear antigen (PCNA; Abcam, ab92552, dilution 1:500), vascular endothelial growth factor (VEGF; Abcam, Cambridge, Cambrigdeshire, UK, ab32152, dilution 1:100), and Monocyte Chemoattractant Protein-1 (MCP-1; Abcam, ab25124, dilution 1:100), and kept in an oven at 37 °C overnight. Subsequently, the slides were hydrated with 0.05M Phosphate-Buffered Saline (PBS) for 5 min at room temperature. After this phase, the sections were incubated and protected from light for 15 min with 50 μL peroxidase. Then, the samples went through the antigenic recovery process, being placed in 10 mmol/L citrate buffer (pH = 6.0) associated with heat during 10 min in a microwave. After the slides cooled, the sections were blocked with 50 µL/section of reagent A from the Invitrogen^®^ kit for 10 min. Except for the slides used as a negative control of the reaction, the primary antibody of interest was added to each cut and diluted in 0.05M PBS, overnight, at 4 °C. After this step, the samples were washed with 0.05M PBS and incubated with anti-Rabbit secondary antibody (Thermo Fisher, Waltham, MA, USA), 31460, dilution 1:400) for 60 min, at room temperature. Immunoactivity was visualized by incubation with Diaminobenzidine (DAB) for 1 min in the dark. The sections were stained with Mayer’s Hematoxylin for 15 s, and finally, the slides were assembled to be analyzed with Nikon DS-Ri1 (Melville, NY, USA). The images were analyzed qualitatively for cell proliferation, blood vessels, and monocytes.

#### 2.2.9. Immunoblotting

The tissue samples on the 3rd, 7th, and 14th days after the lesion were homogenized in a buffer containing 1% Triton X-100, 100 mmol/L Tris (pH 7.4), 10 mmol/L Sodium Pyrophosphate, 100 mmol/L Sodium Fluoride, 10 mmol/L Ethylenediaminetetraacetic Acid (EDTA), 10 mmol/L Sodium Orthovanadate, 2 mmol/L Phenylmethylsulfonyl Fluoride (PMSF), and 0.01 mg/mL Aprotinin, using the Tissuelyzer equipment (Qiagen^®^, Stockah, Germany).

The homogenate was centrifuged at 12,000 rpm at 4 °C for 20 min. Soon after this process, the supernatant was aspirated and its proteins were quantified with the bicinchoninic acid reagent. Then, the samples received Laemmli buffer plus Dithiothreitol (DTT) 200 mmol/L in a 5:1 ratio. After protein quantification, 50 µg of total proteins were submitted to electrophoresis in polyacrylamide gel (SDS-PAGE 12%) in the BioRad minigel equipment with an electrophoresis buffer solution.

After blocking with 5% Molico^®^ powdered milk, the membrane was washed with a basal solution for 15 min and incubated with specific antibodies in a blocking solution with 3% Bovine Serum Albumin (BSA) and kept at 4 °C overnight under gentle stirring. We used the following antibodies: PCNA (Abcam, ab92552), VEGF (Abcam, ab32152), TNF-α (Santa Cruz, H-156, Dallas, TX, USA), and smooth muscle actin (α-SMA) (Santa Cruz), all diluted in a solution of 3% Bovine Serum Albumin (BSA) in the ratio of 1:1000. The next day, the material was washed with a basal solution and incubated with a solution with the secondary antibody (Thermo Fisher, 31460, dilution 1:5000) diluted in 3% powdered milk solution for about 1 h.

Next, about 2 mL of chemiluminescence solution was added in the 1:1 dilution of the reagents of the BioRad^®^ commercial kit for about two minutes. The light emission was detected and captured by the BioRad^®^ photo documentation system (Hercules, CA, USA). The optical density reading was performed using ImageJ^®^. Results were normalized by comparing the expression with β-actin (Cell, 4967L, dilution 1:1000).

#### 2.2.10. Statistical Analysis

For the statistical analysis of the WHR, histomorphometric analysis, birefringence of collagen fibers, protein expression in the Western blot, and the Student’s *t*-test were performed using SPSS Statistics 20.0 (IBM Company New York, NY, USA). A 5% (*p* < 0.05) significance level was adopted.

## 3. Results

### 3.1. Morphological Aspect and Other Relevant Characteristics of the Membranes

**Figure 1:**
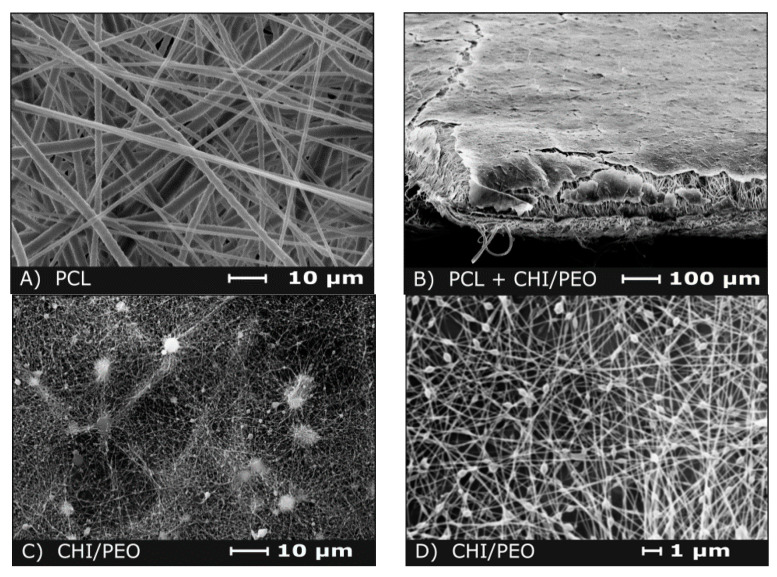
Aspect of the membranes analyzed by SEM. **A**) polycaprolactone (PCL) dressing; **B**) PCL dressing (larger fibers, bottom region) coated with the chitosan (CHI)/poly(ethylene oxide) (PEO) layer (shown as a thin membrane, top region); **C** and **D**) Detail of the structure of the CHI/PEO layer with the bead-on-string morphology.

The membranes morphology was analyzed by SEM (Figure 1), showing a typical structure of interconnected open macropores. There was a significant difference in the diameter of the fibers of PCL and CHI/PEO, as can be seen in Figure 1B and shown more closely in Figure 1A,C. As previously reported, the mean diameter of PCL fibers is around 1.6 ± 0.7 μm, while the diameters of chitosan/PEO fibers are below 200 nm diameter [33].

A bead-on-string morphology was observed for the CHI/PEO fibers, but no major impacts would be expected from it. If desired, such morphology could be avoided by reducing the solution flow rate (but it would lead to an increase in the processing time) or increasing the PEO content in the mixture (lowering the chitosan biological performance contribution).

The mechanical properties of PCL and PCL+CHI/PEO membranes were previously reported by Trinca et al. [33]. Briefly, the mechanical properties of the mats are governed by the PCL layer: tensile strength equal to 1.47 ± 0.09 MPa, Young’s modulus of 10.2 ± 1.4 GPa, and elongation at break above 500% (no break at the experimental conditions). These membranes showed an average thickness of 0.35 ± 0.04 mm, porosity equal to 74 ± 6%, and water vapor permeation rate of 729 ± 4 g/(m²·day).

The morphologies of the single-layer membrane of PCL and double-layered membrane of PCL+CHI/PEO, in which PCL is expected to take the main role of a mechanical support for chitosan-based fibers, are suitable for application as scaffolds and dressings [41,42].

### 3.2. Morphological Analysis of the Lesions

**Figure 2:**
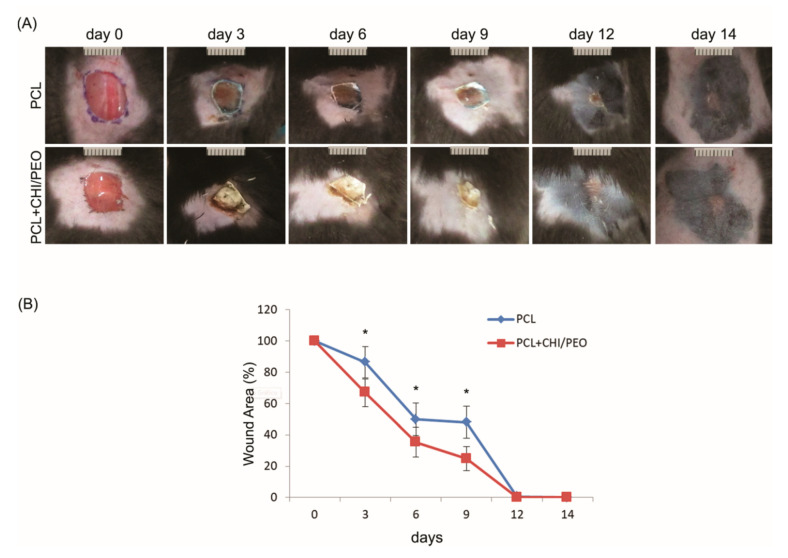
(**A**) Macroscopic appearance of the lesions immediately after the surgical procedure and on the 3, 6, 9th, 12th, and 14th days after contact with the membranes. (**B**) Areas of lesion measured with ImageJ^®^ expressed as a percentage in the same periods mentioned above. The results were expressed as mean ± standard deviation. * *p* < 0.05 indicates statistically significant differences between treatments according to the Student’s *t*-test; (*n* = 8).

As shown in Figure 2, the mice that received topical treatment with PCL+CHI/PEO membrane, compared with those that received treatment with PCL, showed lower WHR on the 3rd, 6th and 9th days after the wounds were made, under macroscopic analysis. All animals showed complete reepithelization after 12 days of treatment.

### 3.3. Histological Analysis

**Figure 3:**
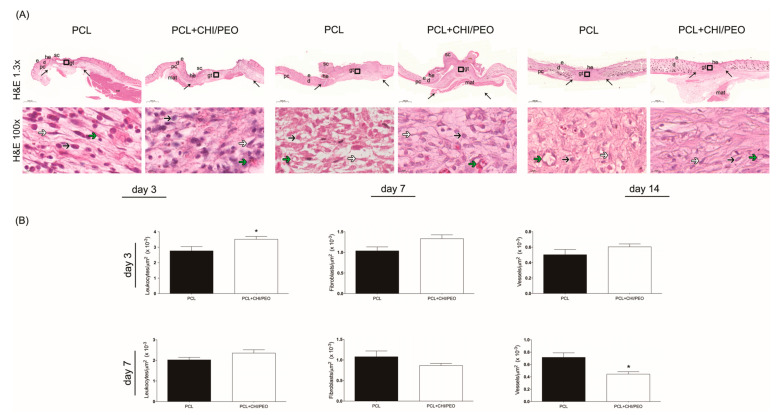
(**A**) Histological analysis of the injured region of the PCL and PCL+CHI/PEO groups on the 3rd, 7th, and 14th day after lesion with Hematoxylin and Eosin stain. The arrows in the upper panels indicate the limits of granulation tissue area and small letters represent: d, dermis; e, epidermis; g, granulation tissue; he, hypertrophic epidermis; sc, scab; pc, *panniculus carnosus* and mat, multilocular adipose tissue. Boxes in the upper panels indicate the area where the regions are shown in higher magnification to illustrate leucocytes (black arrows), fibroblasts (white arrows) and microvessels (green arrows). Objective: 1.3× (upper panels) and 100× (lower panels). (**B**) Graphical representation of the number of inflammatory infiltrates, blood vessels, and fibroblasts in the region of excisional lesions treated topically with PCL and polycaprolactone coated with chitosan and poly(ethylene oxide) (PCL+CHI/PEO) membranes on the 3rd and 7th day after lesion. The results were expressed as mean ± standard deviation. *p < 0.05 indicates statistically significant differences between treatments according to the Student’s *t*-test.

The results of the histological evaluation performed on the 3rd, 7th, and 14th days after the lesion with the H&E stain are shown in Figure 3A. On the 3rd day, the migration of keratinocytes at the edge of the lesion is observed only in the PCL+CHI/PEO group.

On the 7th day, a crust is seen over its area, the hyperplastic newly formed epithelium above the normal line to the adjacent epithelium, and most animals presented complete reepithelization. The lesion area was filled with connective scar tissue with small collagen deposition, of great extension and congested. This scar region, called granulation tissue, presented a high concentration of leukocytes and fibroblasts on the 7th day after the lesion. The dermis adjacent to the lesion and the dermis muscle layer also had leukocytes, showing that the inflammation was not limited to the injured area, but spread around the focus of the initial incision. On the 14th day, the resolution of the inflammatory process is observed, with maturation of the connective tissue in both treatments.

### 3.4. Morphometric Analysis

The morphometric analysis included the counting of inflammatory cells, vessels, and fibroblasts on days 3 and 7 after the lesion. On the 3rd and 7th days, the PCL+CHI/PEO group had a greater number of inflammatory cells compared to the PCL group, with no statistically significant differences in the number of blood vessels and fibroblasts, although there was a reduction tendency in both on the 7th day as showed in Figure 3B.

### 3.5. Immunohistochemical Analysis

**Figure 4:**
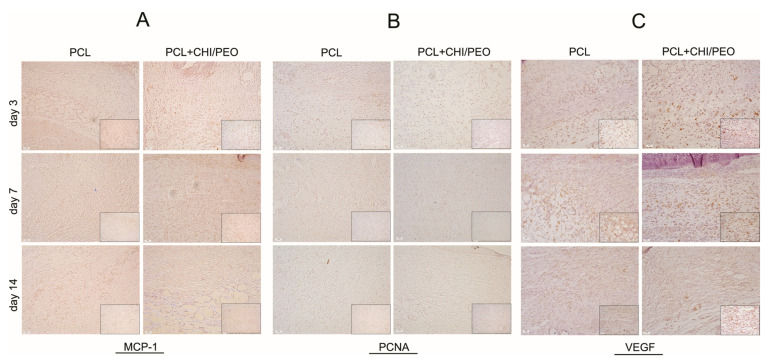
Immunohistochemistry for Monocyte Chemoattractant Protein-1 (MCP-1) (**A**), proliferating cell nuclear antigen (PCNA) (**B**), and vascular endothelial growth factor (VEGF) (**C**) of the areas with excisional lesions treated with PCL or PCL+CHI/PEO membrane on the 3rd, 7th, and 14th day. Objective 20× and 40× (inserts).

Immunolabeling was performed on the 3rd, 7th, and 14th days after the lesion. The immunolabeling of MCP-1, as seen in Figure 4, was more intense in connective tissue, adipose tissue, and around sebaceous glands.

On the 3rd day, the groups had similar markings. On the 7th day, the PCL+CHI/PEO group showed greater immunolabeling, especially close to the epidermis, compared to the 3rd day and to the PCL group on the 7th day. On the 14th day, MCP-1 expression was similar in both groups, remaining similar to the 7th day.

PCNA immunolabeling was more intense in the dermis, close to fibroblasts, and around sebaceous glands. On the 3rd and 7th day after the lesion, immunolabeling was similar in both groups, with increased immunolabeling on the 7th day. On the 14th day, there was a reduction in marking in the groups compared with the other experimental periods (Figure 4).

The immunolabeling of VEGF on the 3rd day was more intense around the brown adipose tissue restricted to the lesion area. On the 7th day, a similar marking was observed in both, but with greater immunolabeling compared to the 3rd day. On the 14th day, there was a reduction in marking in the groups compared with the other experimental days.

### 3.6. Collagen Fiber Analysis

**Figure 5:**
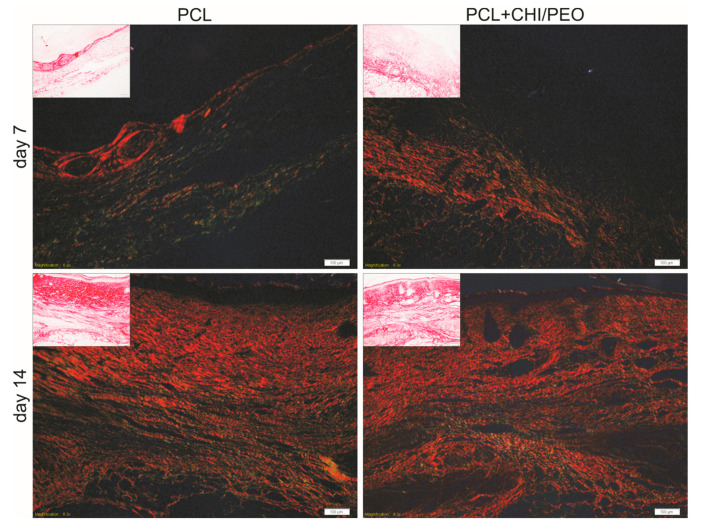
Collagen reorganization and representative results in the skin wound tissue of mice in 7 and 14 days. Collagen fibers from the region of mice excisional lesions treated with PCL or PCL+CHI/PEO membrane on the 7th and 14th experimental day with Sirius Red staining under polarized light and without polarized light (inserts). Objective: 10×.

The qualitative analysis showed that on the 7th day after the lesion, both groups presented collagen deposition, with thin and parallel fibers (Figure 5). On the 14th day, the reconstituted epithelium was no longer thick, and the area where the lesion occurred can be seen due to the absence of hair follicles, sebaceous glands, muscle, and adipose tissue in the PCL and PCL+CHI/PEO groups.

However, the scar area of both groups had a typical appearance, with collagen fibers organized in parallel clusters and covering a more extensive area of the dermis. The quantitative analysis showed no statistical difference between the groups regarding the average density of red and green collagen fibers on the 7th and 14th days after the lesion (data not shown).

### 3.7. Immunoblotting

**Figure 6:**
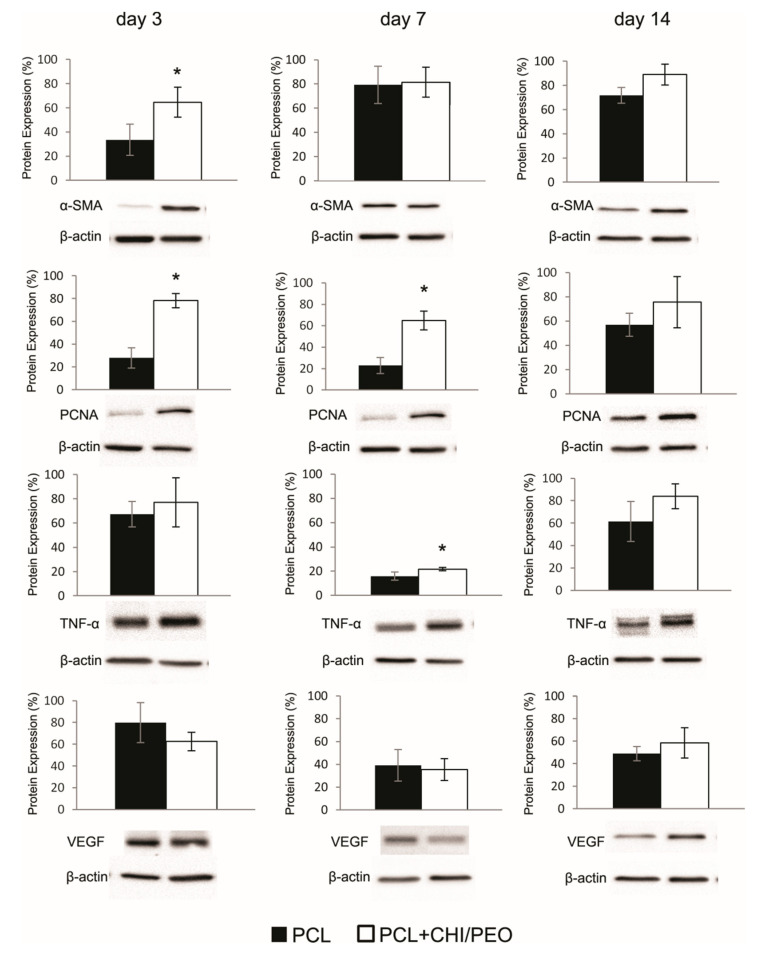
Western blot analysis and densitometric analysis of smooth muscle actin (α-SMA), PCNA, Tumor Necrosis Factor (TNF-α), and VEGF observed in the excision lesions of mice topically treated with PCL or PCL+CHI/PEO membrane on days 3, 7, and 14. The results were expressed as mean ± standard deviation. (*) *p* < 0.05 indicates statistically significant differences between treatments according to the Student’s *t*-test. (*n* = 4–6). Protein expression levels were standardized against the internal β-actin expression levels of each sample.

The results of the immunoblotting analysis are shown in Figure 6. On the 3rd day, the animals in the PCL+CHI/PEO group showed greater expression of α-SMA and PCNA compared with the PCL group. On the 7th day, the PCL+CHI/PEO group had greater expression of PCNA and TNF-α compared with the PCL group. Concerning the expression of VEGF, there was no difference between the groups in the three periods analyzed.

## 4. Discussion

Wounds represent a major burden on health systems and affect the quality of life not only of patients, but also of their families. They are responsible for causing pain, depression, social isolation, and may lead to longer hospitalization periods. For these reasons, the search for new therapeutic products, which may contribute to tissue repair, has been continuous [43,44].

In this study, we compared the performance on skin tissue repair of fibrous electrospun dressings consisting of PCL or PCL+CHI/PEO. All polymers used are biodegradable, biocompatible, and safe. The PCL layer was conceived to simultaneously provide mechanical support to the structure, to act as a lesion protective barrier, and to ultimately function as a scaffold for cell growth. We also included the CHI/PEO blend layer in the formulation to improve the dressing wettability, considering that both chitosan and PEO are more hydrophilic than PCL, and the dressing bioactivity would be improved by the addition of chitosan. These polymers can be effectively used to control bleeding, to promote wound healing, and to improve local collagen concentration. Moreover, chitosan has antibacterial activities [45,46].

Studies have also shown that chitosan favors all stages of the healing process. In the inflammatory phase, this compound can promote the infiltration of polymorphonuclear neutrophils and macrophages [47].

Both membranes, by the histological evaluation of excisional lesions, favored the reepithelization of the skin tissue, suggesting that the addition of chitosan/PEO fibers to the PCL membrane was important for the time of reepithelization of skin tissue in mice. On the other hand, in the morphometric evaluation, PCL+CHI/PEO favored the migration of inflammatory infiltrate on the 3rd and 7th days after the lesion.

Although neutrophils, monocytes, macrophages, and lymphocytes actively participate in the inflammatory response, monocytes and macrophages play an important role in this regulation. These cell types also regulate the reepithelization and remodeling of tissue repair [48], which may explain the greater expression of MCP-1 on the 7th day after lesion in animals treated with PCL+CHI/PEO. Studies show that MCP-1 is one of the inflammatory mediators that activate and attract monocytes and macrophages to the lesion site [49,50], and also that knockout animals for MCP-1 have delayed reepithelization, angiogenesis, and decreased collagen production [51].

In addition to these functions, monocytes and macrophages are important sources of cytokines and growth factors [50]. The production of these substances is essential to stimulate the proliferation, migration, or differentiation of fibroblasts, keratinocytes, and endothelial cells, and for the deposition of the extracellular matrix, reepithelization, and neovascularization [1,52].

The cytokines secreted by monocytes and macrophages include TNF-α, a pro-inflammatory cytokine important for the healing process, but with pleiotropic effects, which makes it take part in other cellular events and not only in the inflammatory phase. It has several effects, such as the chemotaxis and activation of macrophages, favoring angiogenesis, and promoting the formation of an extracellular matrix in the wound by inducing the generation of proteoglycans and fibronectin by fibroblasts [53].

A study [54] that suppressed TNF-α activity with monoclonal antibodies in mouse wounds observed delayed wound closure, greater distance between the *panniculus carnosus* and the edges of the lesion, and decreased inflammatory cells and fibroblasts. In a treatment with bioactive TNF-α, these findings were reversed, showing the importance of TNF-α in tissue repair. In our study, an increase in TNF-α was observed in the three extraction periods, with a statistical difference for the 7th day, suggesting that the membrane modulated the expression of TNF-α, as also previously observed.

The topical treatment with PCL+CHI/PEO membrane did not modulate PCNA immunolabeling in both groups, which is a marker of cell proliferation. On the other hand, when PCNA expression was analyzed in the presence of the PCL+CHI/PEO membrane by immunoblotting, an increase was observed on the 3rd and 7th day after lesion. The same occurred for α-SMA, which is a positive marker for myofibroblasts, on the 3rd day. The differentiation of fibroblasts into myofibroblasts is related to the expression of α-SMA during the healing process [55]; another participation of myofibroblasts in tissue repair is the ability to increase collagen synthesis and the contractile force of the lesion [56]. These findings do not seem to contribute to the results observed for collagen fibers, since they were homogeneous in the PCL and PCL+CHI/PEO groups.

There was no direct effect of PCL+CHI/PEO on the immunolabeling of VEGF or expression of this growth factor. However, studies with chitosan in formulations such as hydrogels, films, and membranes, associated or not with other compounds, have shown promising results as a topical therapy for skin wounds, contributing to the inflammatory phase, the proliferation of fibroblasts, and reorganization of collagen fibers [57,58,59].

In summary, our results show that the CHI/PEO layer incorporated into the PCL membrane did little to improve the tissue repair of excisional lesions in mice compared to the PCL matrix alone. However, several reports in the literature support the positive action of free chitosan or combined with other formulations in tissue repair, such as particles, sponges, hydrogels, or films. Similar observations are reported for PEG [3,46].

The results obtained may be attributed to the small thickness of the deposited CHI/PEO layer (a few micrometers) and its high surface area. This may have led to the rapid hydrolysis of chitosan by lysozyme promoted by the combination of the polysaccharide with the synthetic polymer, which facilitates the access of the enzyme to chitosan, ultimately resulting in a similar behavior between the two biomaterials, which are constituted primarily of PCL.

Since the purpose of this research was to compare the effects of electrospun wound dressings consisting of only PCL with those produced using PCL+CHI/PEO, the results of a control study with untreated animals are not presented herein. This may be a limiting factor because the changes observed in the wounds may not be solely attributed to the differences in the dressing formulations, but experiments run concomitantly have shown that lesions treated only with saline followed the regular course of healing.

We conclude that the PCL+CHI/PEO membrane modulated the inflammatory infiltrate, the expression of MCP-1, PCNA, and α-SMA in excisional lesions in healthy mice, but not enough to favor tissue repair compared with the group treated only with PCL, which can be an important advantage, since it greatly facilitates the process of production and manipulation of the dressing.

Based on the present work, the membranes can be considered suitable for wound dressing applications, and their use could probably be extended to tissues other than the skin. The membranes are easy to prepare, their components are biocompatible and readily available, and it is possible to incorporate relevant bioactive agents in them, such as antibiotics, drugs with anti-inflammatory activity, and growth factors to further expand their plethora of applications.
