# Correction: Zanchetta et al. Effects of Electrospun Fibrous Membranes of PolyCaprolactone and Chitosan/Poly(Ethylene Oxide) on Mouse Acute Skin Lesions. *Polymers* 2020, *12*, 1580

**DOI:** 10.3390/polym16121737

**Published:** 2024-06-19

**Authors:** Flávia Cristina Zanchetta, Rafael Bergamo Trinca, Juliany Lino Gomes Silva, Jéssica da Silva Cunha Breder, Thiago Anselmo Cantarutti, Sílvio Roberto Consonni, Ângela Maria Moraes, Eliana Pereira de Araújo, Mario José Abdalla Saad, Gary G. Adams, Maria Helena Melo Lima

**Affiliations:** 1 School of Nursing, University of Campinas, Campinas CEP 13083887, Brazil; flaviac.zanchetta@gmail.com (F.C.Z.); julianyl@unicamp.br (J.L.G.S.); jecunha.silva@gmail.com (J.d.S.C.B.); biocantarutti@gmail.com (T.A.C.); pa.eliana@gmail.com (E.P.d.A.); 2 Department of Engineering of Materials and of Bioprocess, School of Chemical Engineering, University of Campinas, Campinas CEP 13083852, Brazil; rafaeltrinca@gmail.com (R.B.T.); ammoraes@unicamp.br (Â.M.M.); 3 Department of Biochemistry and Tissue Biology, Institute of Biology, University of Campinas, Campinas CEP 13083970, Brazil; consonni@unicamp.br; 4 Department of Internal Medicine, University of Campinas, Campinas CEP 13083887, Brazil; msaad@fcm.unicamp.br; 5 School of Health Sciences, Faculty of Medicine, The University of Nottingham, C Floor, South Block Link, Queen’s Medical Centre, Nottingham NG7 2HA, UK

## Error in Figure

In the original publication, the authors claimed that Figure 6 reporting Western blot data was erroneous as published. Specifically, the PCNA band on day 3 was found to be duplicated with PCNA band on day 7. We replaced the image from PCNA on day 3. The corrected Figure 6 (PCNA bands) appears below. The authors state that the scientific conclusions are unaffected. This correction was approved by the Academic Editor. The original publication has also been updated [1].

## Figures and Tables

**Figure 6:**
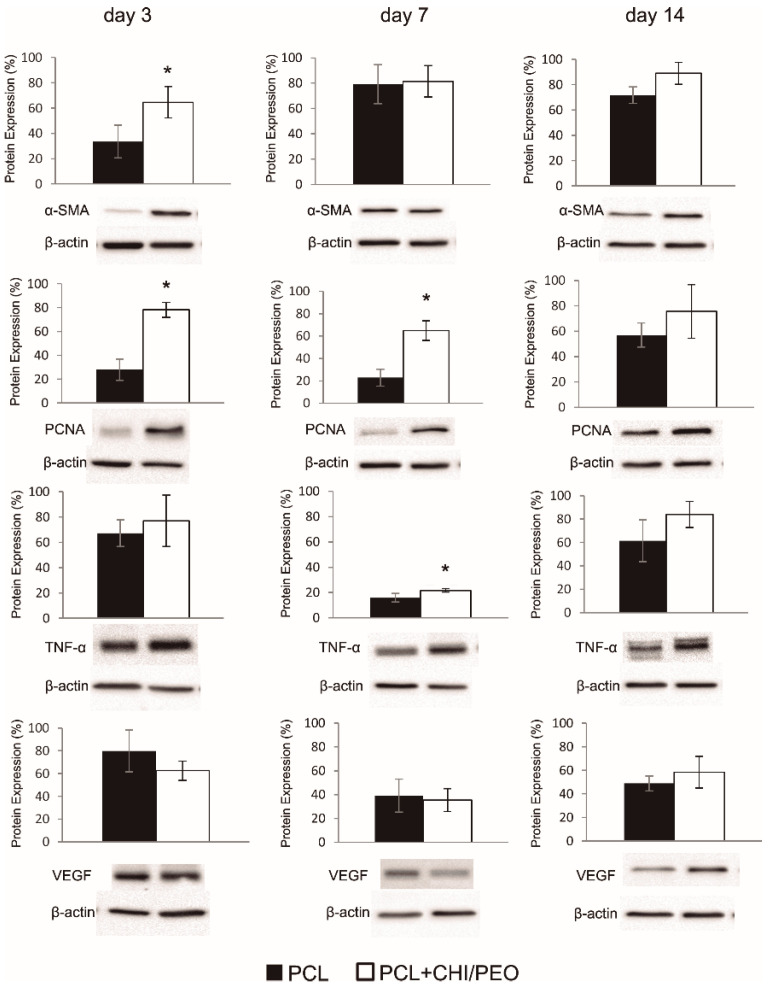
Western blot analysis and densitometric analysis of smooth muscle actin (α-SMA), PCNA, Tumor Necrosis Factor (TNF-α), and VEGF observed in the excision lesions of mice topically treated with PCL or PCL+CHI/PEO membrane on days 3, 7, and 14. The results were expressed as mean ± standard deviation. (*) *p* < 0.05 indicates statistically significant differences between treatments according to the Student’s *t*-test. (*n* = 4–6). Protein expression levels were standardized against the internal β-actin expression levels of each sample.
